# One-Pot Synthesis of HRP&SA/ZIF-8 Nanocomposite and Its Application in the Detection of Insecticidal Crystalline Protein Cry1Ab

**DOI:** 10.3390/nano12152679

**Published:** 2022-08-04

**Authors:** Ranfeng Ye, Hao Chen, He Li

**Affiliations:** 1 College of Science, Huazhong Agricultural University, Wuhan 430070, China; 2 College of Optoelectronics Technology, Chengdu University of Information Technology, Chengdu 610225, China

**Keywords:** zeolitic imidazolate framework-8, immunoassay, Bt protein, Cry1Ab

## Abstract

This study reported the functionality integration of zeolitic imidazolate framework-8 (ZIF-8) with horseradish peroxidase (HRP) and streptavidin (SA) for the synthesis of a HRP&SA/ZIF-8 nanocomposite through one-pot coprecipitation. The synthesized HRP&SA/ZIF-8 nanocomposite was then employed as the ideal signal tag for application in the enzyme-linked immunosorbent assay (ELISA) and exhibited excellent sensitivity, selectivity and accuracy in the detection of insecticidal crystalline (Cry) protein Cry1Ab as a transgenic biomarker with a detection limit of 4.8 pg/mL. This proposed method provides a new way for the detection of transgenic biomarkers in food and may inspire further integration of a variety of biomolecules into ZIF-8 for applications ranging from biosensing, biomedicine, and catalysis to energy.

## 1. Introduction

*Bacillus thuringiensis* (Bt) is an important Gram-positive bacillus that can be applied as an efficient insecticide, owing to its ability to secrete insecticidal crystal proteins (Bt proteins) which include two main parasporal toxins, crystalline (Cry) and cytolytic (Cyt) toxins [1,2]. With the development of transgenic technology and increased demand for food crop production, the Bt gene can be implanted into targeted crops by transgenic technology to express the Bt protein to effectively protect agricultural food crops such as maize, soybean and rice against crop insects [3,4,5]. However, although Bt proteins have a strong killing effect on crop insects, the nontarget toxicity of Bt proteins on other creatures also gains much concern from the public [6,7]. For a better response and regulation of transgenic products and food safety, the determination of the content of Bt protein in transgenic food crops or related environmental samples is particularly important and must be the first step.

At present, there are mainly two kinds of methods for detecting Bt protein. One is nucleic-acid-based detection methods, such as polymerase chain reaction and microarray [8,9]. Such methods are relatively complex in their operation process and not suitable for large-scale rapid detection on site, which usually requires professional instruments and equipment. The other is protein-based detection methods, such as enzyme-linked immunosorbent assay (ELISA) and immunochromatography assay (ICA) that are suitable for the detection of large quantities of samples due to their relatively simple operation and cost-efficiency [10,11]. However, the traditional ELISA and ICA method, due to the limited number of enzyme molecules bound to the analyte, often suffer from the limitation of its sensitivity and cannot meet the requirement of detection of a low content of residual Bt protein in the environment [12,13].

To overcome this challenge, nanostructure-based materials have gained much interest owing to the possibility of functionalized nanomaterials to achieve specific properties. With the high specific surface area and biocompatibility, nanomaterials are considered to be the appropriate carrier for immobilizing sufficient enzyme molecules to achieve signal amplification [14,15]. Usually, immobilization strategies work through adsorption or covalent attachment to integrate enzymes on the presynthesized nanomaterials [16]. However, these strategies have the disadvantages of easy leaching or activity loss of enzymes and complicated operation and purification processes [17]. Among the many nanomaterials, zeolitic imidazolate framework-8 (ZIF-8) is the subclass of metal–organic frameworks (MOFs) and has received much attention for its high thermal and chemical stability and synthesis under mild biocompatible conditions [18,19,20,21]. These properties enable ZIF-8 to become an ideal candidate as the immobilization carrier to address the issue mentioned above. For example, through one-pot coprecipitation, Lyu et al. synthesized cytochrome c (cyt c)/ZIF-8 nanocomposite that was 10-fold enhanced in its activity compared with free cyt c, which exhibited excellent performance in the detection of explosive organic peroxides [22]. Wang et al. prepared glucose oxidase (GOx) and NiPd-nanoparticles-coencapsulated ZIF-8 through the coprecipitation method, which was successfully applied in the rapid sensing of glucose and showed high electrocatalytic activity for the oxygen reduction reaction [23]. Ricco et al. used HRP and iron oxide magnetic nanoparticles to construct magnetically responsive HRP@ZIF-8 with one-pot synthesis, which provided a new platform for reusable biocatalysts [24]. These studies revealed the great promise of ZIF-8 in biocatalysis and biosensing. 

Until now, the coimmobilization of the enzyme and recognition protein in ZIF-8 for application in the colorimetric immunoassay, especially for the detection of Bt protein, has been rarely explored. Thus, in this study, the horseradish peroxidase as the enzyme and streptavidin as the recognition protein were employed and coimmobilized in the ZIF-8 to prepare an HRP&SA/ZIF-8 nanocomposite integrating the bifunction of signal amplification and biorecognition. The morphology, size and structure of the synthesized HRP&SA/ZIF-8 nanocomposite were characterized. Under optimized conditions, the proposed HRP&SA/ZIF-8 nanocomposite then served as the ideal signal label in ELISA for application in the detection of the Bt protein Cry1Ab as a transgenic biomarker. The sensitivity, selectivity and accuracy of this method were also presented and discussed in detail.

## 2. Material and Methods 

### 2.1. Materials and Reagents 

Streptavidin (SA), hemoglobin, horseradish peroxidase (HRP), bovine serum albumin (BSA), 3,3′,5,5′-tetramethylbenzidine (TMB) and polyvinyl pyrrolidone (PVP) were obtained from Sigma-Aldrich. 2-methylimidazole, zinc nitrate (Zn (NO_3_)_2_), tween-20, sulfuric acid (H_2_SO_4_), potassium dihydrogen phosphate (KH_2_PO_4_), dibasic sodium phosphate (Na_2_HPO_4_), potassium chloride (KCl) and sodium chloride (NaCl) were purchased from Sinopharm Chemical Reagent Co., Ltd. (Shanghai, China). 96-well ELISA plates were bought from Corning-Costar. CP4-EPSPS, Cry1Ab, Cry1e, biotin-antibody and monoclonal antibody to Cry1Ab were acquired from Youlong Biotech Co., Ltd. (Shanghai, China). All chemical reagents were used as received without further purification. 

### 2.2. Instruments

The scanning electron microscope (SEM) and transmission electron microscopy (TEM) images were obtained with the NTC JSM-6390LV SEM system and Hitachi H-7650 TEM system, respectively. The X-ray diffraction (XRD) analysis was conducted on the Rigaku Miniflex 600 system. The solution in 96-well ELISA plates for the absorbance determination was performed with the SpectraMax i3 multifunctional system. 

### 2.3. Synthesis and Characterization of HRP&SA/ZIF-8 Nanocomposite

In a typical synthesis, 0.1 mL 0.30 M Zn (NO_3_)_2_, 5 μL 1 mg/mL SA, and 5 μL 1 mg/mL HRP were added to 1 mL 1.2 M 2-methylimidazole at room temperature and the mixture was stirred for 30 min. The precipitate was collected by centrifugation at 7000 rpm for 15 min and then washed with deionized water 3 times. The obtained product was redispersed in 1 mL phosphate-buffered saline (PBS) buffer solution and stored at 4 °C before use. For the SEM and TEM observation, a drop of the as-prepared nanocomposites was dried on the conductive adhesive and carbon grid, respectively. For the XRD, the as-prepared HRP&SA/ZIF-8 nanocomposite was processed with the vacuum freeze-drying treatment before the analysis. The simulated XRD pattern of ZIF-8 was analyzed by Mercury software version 2022.2.0 (Cambridge Crystallographic Data Centre, Cambridge, UK).

### 2.4. Optimization of the Experimental Conditions

To obtain the best performance, the optimal total amount of HRP and SA (0.02, 0.03, and 0.04 mg), mass ratio of HRP to SA (1:1, 3:1, 5:1, and 7:1), pH value (5.5, 6.0, 6.5, 7.0, and 7.5), the dilution ratio of synthesized nanocomposite (1:5, 1:10, 1:15, and 1:20) for application in the detection and incubation time of the synthesized nanocomposite (15, 20, 25, 30, and 35 min), respectively, were determined.

### 2.5. Procedure for Cry1Ab Detection

The procedure for detection was similar to the traditional ELISA and the ELISA plate was carefully washed by PBS 3 times after each step. Briefly, the ELISA plate was firstly coated with 100 μL 1 mg/mL monoclonal antibody to Cry1Ab at 4 °C overnight and then incubated with 10 mg/mL BSA for 1 h blocking. The standard Cry1Ab solutions with different concentrations were added to the plate for 1 h incubation. To form the antigen–antibody sandwich structure, the biotin–antibody to Cry1Ab (100 μL 200 ng/mL) was added to the plate for 1 h incubation. Next, the 100 μL synthesized HRP&SA/ZIF-8 nanocomposite with appropriate dilution was added to the plate and incubated for 20 min. The HRP substrate solution was then added to incubate for 15 min. The reaction was stopped by adding 100 μL per well of 1 M H_2_SO_4_ and the absorbance of the resulting solution was detected by using a microplate reader (SpectraMax i3 system) at 450 nm. 

### 2.6. Selectivity and Recovery Experiment

To investigate the selectivity of this method, the three interferences of hemoglobin, CP4-EPSPS and Cry1e, and the mixture of Cry1Ab and the three interference analytes (each of them at the same final concentration of 5 ng/mL) were detected. To study the recovery of the Cry1Ab, the non-transgenic rice leaves (0.05 g) were carefully ground; then, the extract solution (2 mL phosphate-buffered saline with tween-20 (PBST) containing 1% PVP) was added to dissolve the ground sample and the standard Cry1Ab added to make the final concentrations of 0 ng/mL, 1 ng/mL, 4 ng/mL and 8 ng/mL, which were determined based on the standard curve.

## 3. Results and Discussion

### 3.1. Principle of Nanocomposites’ Preparation and Detection

**Scheme 1:**
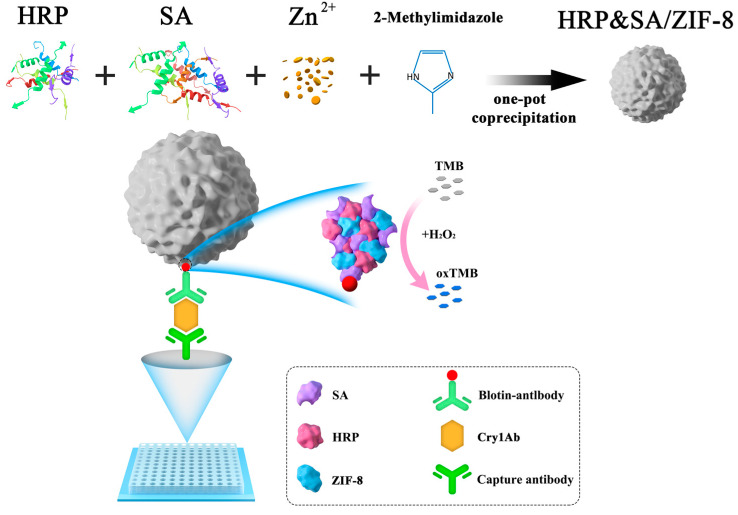
Illustration of the preparation process for HRP&SA/ZIF-8 nanocomposite and its application in the detection of Cry1Ab.

The principle of preparation of the HRP&SA/ZIF-8 nanocomposites and their application in ELISA for detecting Bt protein Cry1Ab are illustrated in Scheme 1. Both the HRP and SA as the protein were encapsulated into ZIF-8 by the biomineralization process that organic templates (protein and 2-methylimidazole) regulated the nucleation and growth of inorganic materials at the organic/inorganic interface to control the morphology and property of the organic/inorganic nanocomposite. This phenomenon was due to the fact that the amino group in the HRP and SA backbone can bind with Zn^2+^. Additionally, 2-methylimidazole can coordinate with Zn^2+^ to form ZIF-8 [25]. This demonstrated the formation of ZIF-8 along with the encapsulation of HRP and SA via one-step coprecipitation. Zn^2+^ is a linker for loading sufficient HRP for signal amplification and the appropriate amount of SA for biorecognition. The as-prepared HRP&SA/ZIF-8 nanocomposites were then applied as the signal label in ELISA for the sensitive detection of Cry1Ab. The monoclonal antibody, biotin–antibody and the antigen of Cry1Ab were to form the sandwich complex. Next, the HRP&SA/ZIF-8 nanocomposites were employed and responsible for signal amplification, which were bound to biotin–antibody through biotin–streptavidin interaction. After the reaction was finished, the absorbance could be detected at 450 nm and was proportional to the concentration of Cry1Ab, which can establish a standard curve for the quantitative detection of Cry1Ab.

### 3.2. Characterization of HRP&SA/ZIF-8 Nanocomposites

**Figure 1:**
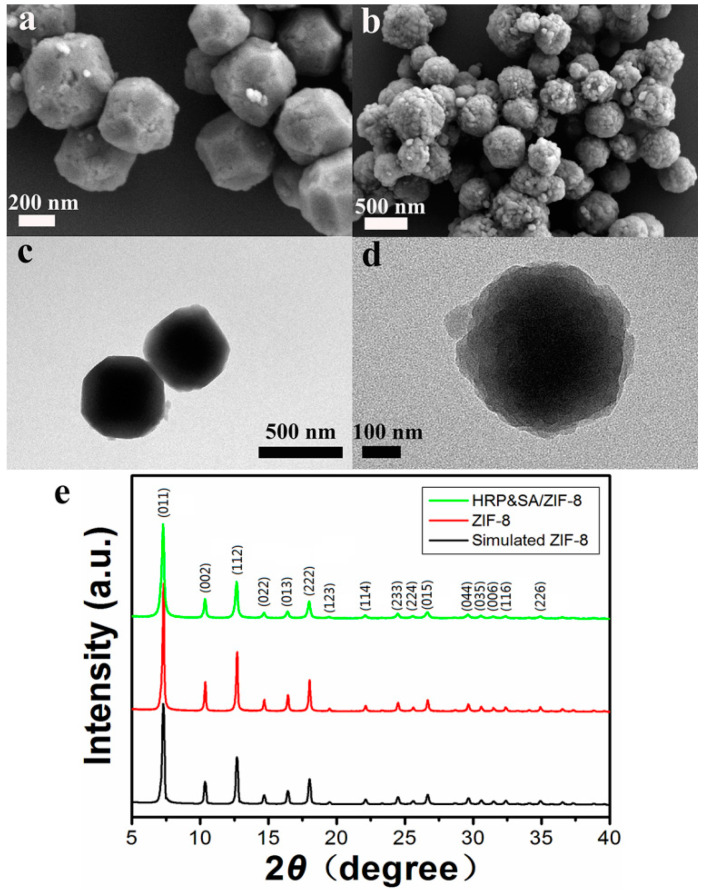
(**a**) SEM image of ZIF-8; (**b**) SEM image of HRP&SA/ZIF-8 nanocomposites; (**c**) TEM image of ZIF-8; (**d**) TEM image of HRP&SA/ZIF-8 nanocomposites; (**e**) XRD pattern of HRP&SA/ZIF-8 nanocomposites.

As shown in Figure 1, the morphology and size of HRP&SA/ZIF-8 nanocomposites were characterized by SEM and TEM. The pure ZIF-8 crystal without encapsulation of HRP and SA was polyhedral with a smooth surface (Figure 1a,c) while the HRP&SA/ZIF-8 nanocomposites displayed a spheroidal structure with a rough surface and a similar size of ~500 nm (Figure 1b,d), which may be due to the encapsulation of HRP and SA into ZIF-8. The X-ray diffraction (XRD) analysis was also conducted to further verify the structure of ZIF-8. Both the XRD pattern of pure ZIF-8 and HRP&SA/ZIF-8 nanocomposites agreed well with the simulated XRD pattern (Figure 1e), indicating that the encapsulation of HRP and SA had no impact on the crystal structure of ZIF-8 and HRP&SA/ZIF-8 nanocomposites were successfully synthesized.

### 3.3. Optimization for the Detection

**Figure 2:**
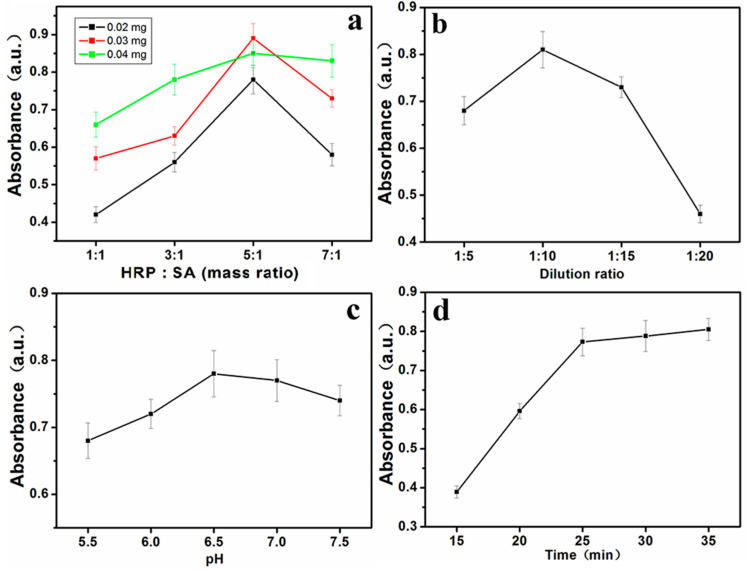
Detection of Cry1Ab with different amounts of HRP and SA. (**a**), at different dilution ratio (**b**), pH values (**c**) and incubation times (**d**) for HRP&SA/ZIF-8 nanocomposites’ binding.

To achieve improved performance for this assay, several detection conditions were optimized (Figure 2). The amount and proportion of HRP and SA encapsulated into ZIF-8 directly determined the signal level and efficiency binding to the analyte; hence, they affected the sensitivity of this method. The 0.03 mg total amount of HRP and SA with the mass ratio of 5:1 can obtain the optimum signal level (Figure 2a). The higher proportion of HRP cannot acquire a higher signal level; this may result from the lack of sufficient SA to ensure the efficient binding of HRP&SA/ZIF-8 nanocomposites. The optimum amount of as-prepared HRP&SA/ZIF-8 nanocomposites was also studied. The maximum absorbance signal occurred when the as-prepared HRP&SA/ZIF-8 nanocomposites in 1 mL PBS stock solution were diluted 10 times for the detection (Figure 2b). 100 μL of the 10-times-diluted stock solution was taken for a single detection in a well of an ELISA plate; therefore, the as-prepared HRP&SA/ZIF-8 nanocomposite could be afforded to the detection of 100 samples of Cry1Ab and the maximum 96 samples of Cry1Ab could be determined in one measurement by using the 96-well ELISA plate. With the increase in the dilution factor of the HRP&SA/ZIF-8 nanocomposites, the absorbance value gradually decreased, which may be due to the decrease in the total amount of HRP in the HRP&SA/ZIF-8 nanocomposites involved in the catalytic reaction. Moreover, the optimum pH and incubation time were also investigated. The optimum signal level was achieved at pH 6.5 (Figure 2c). The higher or lower pH value may not be the best condition for HRP&SA/ZIF-8 nanocomposites binding or HRP catalysis. The optimum incubation time for HRP&SA/ZIF-8 nanocomposites binding was 25 min. The extended period of incubation time could not acquire the significantly higher absorbance signal (Figure 2d). Therefore, pH 6.5 and 25 min of incubation were chosen for HRP&SA/ZIF-8 nanocomposites binding.

### 3.4. Detection of Cry1Ab Using HRP&SA/ZIF-8 Nanocomposite

**Figure 3:**
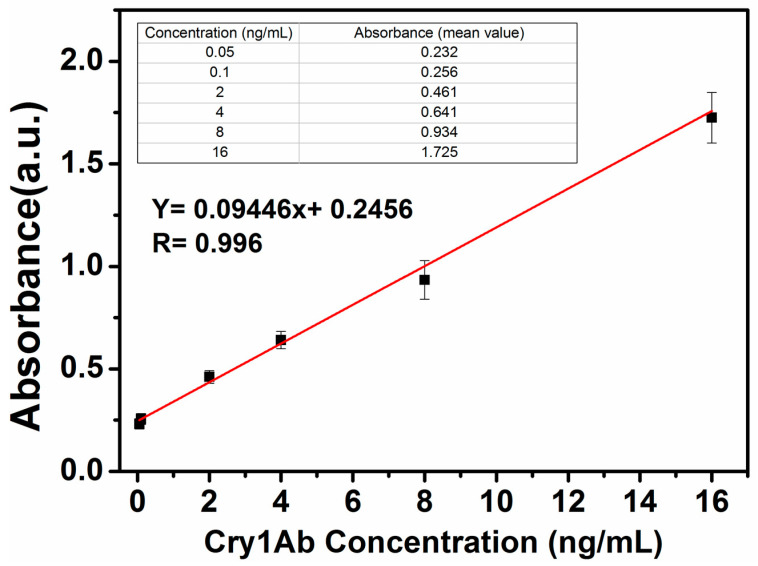
Calibration curve for detection of Cry1Ab at different concentrations.

**Figure 4:**
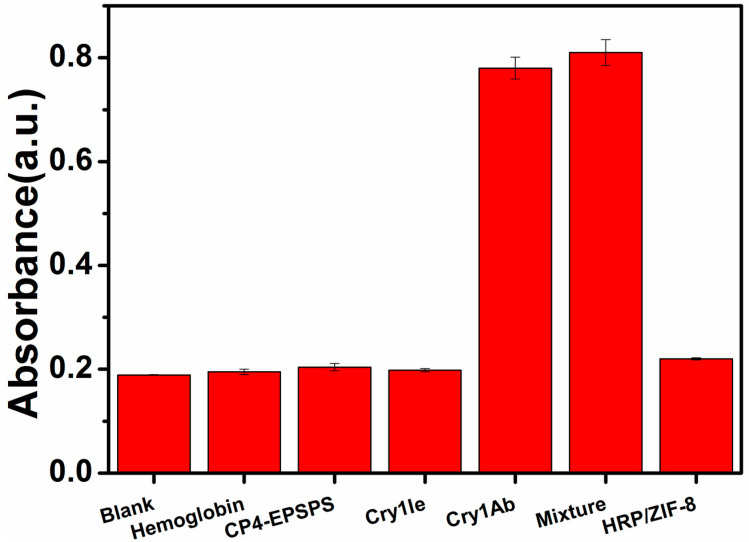
Selectivity of the proposed method.

The detection of the Cry1Ab was based on the traditional sandwich immunoassay. The Cry1Ab was firstly captured by the monoclonal antibodies immobilized on the ELISA plate wells and then they formed a sandwich complex with a biotinylated antibody. The HRP and SA in the HRP&SA/ZIF-8 nanocomposites performed the function of signal amplification and biorecognition, respectively. When the HRP&SA/ZIF-8 nanocomposites finished the binding by biotin-SA interaction, the HRP then catalyzed its substrate to produce a colored solution, which provided an absorbance signal for the quantitative detection of Cry1Ab. Under optimized conditions, a calibration curve was obtained and the range of linearity was from 0.05 to 16 ng, with a detection limit of 4.8 pg/mL according to the 3σ criterion (Figure 3). The formula for calculating the limit of detection (LOD) is as follows: LOD = 3σ/s, where σ was the standard deviation of blank samples’ measurement, and s was the slope of the calibration plot [26]. The result showed that the proposed method showed better performance in its sensitivity, compared with other immunoassay-based methods in Table 1, including the electrochemical, chemiluminescent, immunochromatographic, electrochemiluminescent and impedimetric immunoassay. To further evaluate the selectivity of this method, hemoglobin and the two transgenic-related proteins CP4-EPSPS and Cry1e as the interference analytes were detected. A relatively strong signal absorbance was obtained in the presence of Cry1Ab while a faint absorbance signal was observed for the detection of the other three interference analytes (Figure 4), indicating that the proposed method exhibited a good specificity for Cry1Ab against the selected interfering substances. To verify whether the various sizes of the as-prepared HRP&SA/ZIF-8 nanocomposite affected the proposed analytical method, the precision and reproducibility of this method were also investigated. The proposed method showed a very good precision as evidenced by the low relative standard deviation of 0.96% (n = 8) of eight consecutive measurements of 10 ng/mL Cry1Ab. Additionally, there was a very good reproducibility as evidenced by the low relative standard deviation of 3.83% (n = 5) of five measurements of 10 ng/mL Cry1Ab using the HRP&SA/ZIF-8 nanocomposite prepared at five different times. Thus, the size of the HRP&SA/ZIF-8 nanocomposite prepared in this study had little impact on the proposed analytical method. Moreover, to verify the accuracy and feasibility of this method, the recovery experiments were adopted. The Cry1Ab at various concentrations (including 0, 1, 4, and 8 ng/mL) was added into the spiked non-transgenic rice leaf samples. The recoveries of Cry1Ab were determined to be from 92.0% to 104.3% (Table 2), suggesting good applicability and reliability of the present method.

## 4. Conclusions

In summary, this study demonstrated a one-pot coprecipitation method for the simple and efficient encapsulation of HRP and SA into ZIF-8 to prepare a bifunctional HRP&SA/ZIF-8 nanocomposite without a tedious synthesis and purification process. The HRP&SA/ZIF-8 nanocomposite displayed a spheroidal structure with a rough surface and a size of ~500 nm. The HRP&SA/ZIF-8 nanocomposite was applied in the detection of the protein Cry1Ab as a transgenic biomarker and experiment conditions were carefully optimized (a. the 0.03 mg total amount of HRP and SA with a mass ratio of 5:1; b. the dilution factor of HRP&SA/ZIF-8 nanocomposites for application was 10; c. the pH value and incubation time were 6.5 and 25 min, respectively, for HRP&SA/ZIF-8 nanocomposites’ binding). Under optimized conditions, the proposed method exhibited excellent sensitivity, selectivity and accuracy in the detection of Cry1Ab with a detection limit of 4.8 pg/mL, providing a new way for the determination of the transgenic biomarker in food. This method may inspire further various integrations of biomolecules into ZIF-8 and holds great promise in applications extending from biosensing, biomedicine, and catalysis to energy. 

## Figures and Tables

**Table 1:** Comparison of different immunoassays for Cry1Ab detection.

Methods	Linear Range ng/mL	LOD ng/mL	Reference
Electrochemical immunoassay	0–1000	0.07	[27]
Immunochromatographic assay	-	100	[28]
Chemiluminescent immunoassay	10.49–307.1	6.45	[29]
Electrochemiluminescent immunoassay	0.010–1.0	0.03	[30]
Impedimetric immunoassay	1–10	0.37	[31]
This work	0.05–16	0.0048	This work

LOD: Limit of detection.

**Table 2:** Recoveries of Cry1Ab.

Cry1Ab Added (ng/mL)	Cry1Ab Found (ng/mL)	Recovery (%)
0	not been found	-
1	0.92 ± 0.05	92.0 ± 5.0
4	4.17 ± 0.38	104.3 ± 9.5
8	7.46 ± 0.61	93.4 ± 7.6

## Data Availability

The data presented in this study are available in this article.
